# Comparison of machine learning approaches for positive airway pressure adherence prediction in a veteran cohort

**DOI:** 10.3389/frsle.2024.1278086

**Published:** 2024-02-16

**Authors:** Anna M. May, Jarrod E. Dalton

**Affiliations:** ^1^Geriatric Research Education and Clinical Center and Sleep Medicine Section, Louis Stokes Cleveland VA Medical Center, Cleveland, OH, United States; ^2^School of Medicine, Case Western Reserve University, Cleveland, OH, United States; ^3^Department of Quantitative Sciences, Lerner Research Institute Cleveland Clinic, Cleveland, OH, United States

**Keywords:** sleep apnea, machine learning, adherence, positive airway pressure, compliance

## Abstract

**Background:**

Adherence to positive airway pressure (PAP) therapy for sleep apnea is suboptimal, particularly in the veteran population. Accurately identifying those best suited for other therapy or additional interventions may improve adherence. We evaluated various machine learning algorithms to predict 90-day adherence.

**Methods:**

The cohort of VA Northeast Ohio Health Care system patients who were issued a PAP machine (January 1, 2010–June 30, 2015) had demographics, comorbidities, and medications at the time of polysomnography obtained from the electronic health record. The data were split 60:20:20 into training, calibration, and validation data sets, with no use of validation data for model development. We constructed models for the first 90-day adherence period (% nights ≥4 h use) using the following algorithms: linear regression, least absolute shrinkage and selection operator, elastic net, ridge regression, gradient boosted machines, support vector machine regression, Bayes-based models, and neural nets. Prediction performance was evaluated in the validation data set using root mean square error (RMSE).

**Results:**

The 5,047 participants were 38.3 ± 11.9 years old, and 96.1% male, with 36.8% having coronary artery disease and 52.6% with depression. The median adherence was 36.7% (interquartile range: 0%, 86.7%). The gradient boosted machine was superior to other machine learning techniques (RMSE 37.2). However, the performance was similar and not clinically useful for all models without 30-day data. The 30-day PAP data and using raw diagnoses and medications (vs. grouping by type) improved the RMSE to 24.27.

**Conclusion:**

Comparing multiple prediction algorithms using electronic medical record information, we found that none has clinically meaningful performance. Better adherence predictive measures may offer opportunities for personalized tailoring of interventions.

## 1 Introduction

Obstructive sleep apnea (OSA) affects an estimated 26% of the U.S. population and 47% of veterans with sleep disorders (Peppard et al., 2013; Alexander et al., 2016). OSA causes daytime dysfunction, decreased quality of life, and increased rates of morbidity and mortality, particularly in the veteran population (Guggisberg et al., 2007; Alexander et al., 2016).

Consistent use of positive airway pressure (PAP) therapy, the mainstay of therapy, is effective in reversing OSA pathophysiology. PAP therapy improves blood pressure control, cardiac remodeling, measures of cardiac electrophysiological derangement, and atrial fibrillation recurrence after ablation and cardioversion (Bonsignore et al., 2002; Baranchuk, 2012; Colish et al., 2012; Baranchuk et al., 2013; Gottlieb et al., 2014; Li et al., 2014; Campos-Rodriguez et al., 2017). Treatment impacts patient-centered outcomes, including improved sleep quality, decreased daytime sleepiness, and improved quality of life. Perhaps most impressive, OSA therapy decreased overall health care resource utilization and costs in Canada, a country with nationalized health care, making the lack of health care access unlikely to account for the effect (Albarrak et al., 2005). Taken *in toto*, therapy for OSA not only decreases morbidity but may also decrease health care costs.

OSA therapy is effective. However, adherence to PAP therapy is low-−24% stopped therapy within 3 months, with another 23% being non-adherent with therapy after 1 year (Aloia et al., 2008). Long known to be a problem, adherence has not improved substantially over the past 20 years, despite attempts of intensive interventions (Rotenberg et al., 2016). Patients, providers, and the health care system would reap enhanced efficiency and cost savings by identifying and focusing on those most likely to become non-adherent to therapy.

Although many studies describe associations between patient characteristics or sleep study data and adherence, to our knowledge, no predictive model has been developed. Improving prediction could guide resource allocation and improve outcomes. We developed, validated, and compared several machine learning algorithms to predict adherence within the first 90 days (percentage nights used at least 4 h a night). We hypothesize that screening for non-adherence can be developed using a subset of commonly collected electronic medical record (EMR) variables. We additionally hypothesize that comorbidities, particularly cardiac and psychiatric comorbidities, contribute most to adherence prediction model performance.

## 2 Methods

### 2.1 Participants and study design

**Figure 1:**
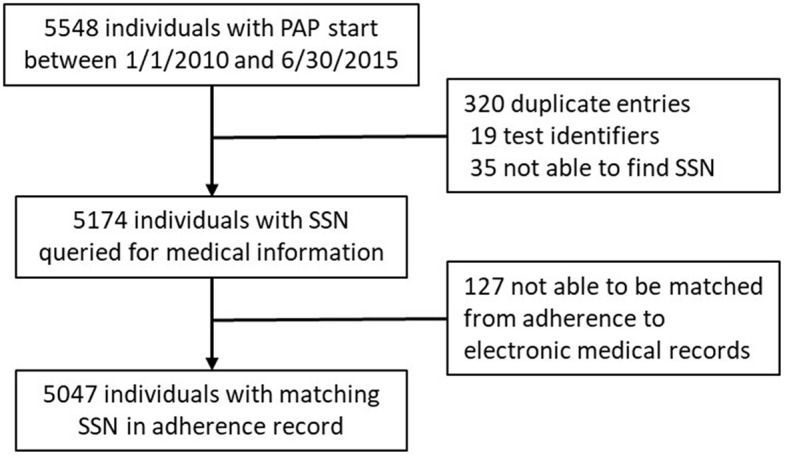
Study flow: recruitment, attrition, and retention.

This retrospective cohort study evaluated adult veteran sleep medicine clinic patients of the VA (Veterans Administration) Northeast Ohio Health Care system. There were 5,548 PAP adherence monitoring records started between January 1, 2010, and June 30, 2015 (Figure 1). Of the 5,174 unique records, 5,047 were able to be matched with VA electronic medical records. The VA Northeast Ohio Health Care system institutional review board found this retrospective study exempt from review.

### 2.2 PAP adherence

All individuals with records of PAP disbursement had ResMed© PAP machines with electronic modems. Adherence data were automatically downloaded every morning to EncoreAnywhere ©. Adherence was defined as the percentage use of at least 4 h a day in the first 90-day period after their PAP disbursement. In addition, we collected 30-day adherence and efficacy metrics, including the percentage of days used more than 4 h/night, mean and 90th percentile pressure, residual apnea–hypopnea index, residual central apnea index, Hunter–Cheyne–Stokes respiration, and leak.

### 2.3 Other measures

All other variables were obtained from the electronic medical record from the time of PAP disbursement or, if not available at that time, the time closest to PAP disbursement. Height and weight were used to compute the body mass index (kg/m^2^). Race was categorized as white, black, or other. Participants' past medical history (obesity, cardiac disease, psychiatric diagnoses, other sleep disorders, diabetes, chronic kidney disease, dementia, stroke, liver disease, pain syndromes, alcohol abuse, and tobacco use) and medications (benzodiazepines, benzodiazepine receptor agonists, other sleep medications, tricyclic antidepressants, antidepressants, antipsychotics, mood stabilizers, α2δ ligands, and stimulants) were obtained. Cardiac disease was defined as a diagnosis of arrhythmia, heart failure, and/or coronary artery disease. Psychiatric disease was defined as schizophrenia, psychosis disorders, depression, posttraumatic stress disorder (PTSD), bipolar disorder, anxiety, and obsessive-compulsive disorder. Other sleep disorders included insomnia, hypersomnia, hypoventilation, circadian rhythm disorders, parasomnia, movement disorders, and narcolepsy. Other sleep medications included ramelteon, diphenhydramine, melatonin, doxepin, mirtazapine, and trazodone. Antidepressants included serotonin reuptake inhibitors, serotonin norepinephrine reuptake inhibitors, mirtazapine, trazodone, and nefazodone. Stimulants included modafinil, armodafinil, methylphenidate, and dextroamphetamine.

## 3 Statistical analyses

Participant characteristics were summarized as mean ± standard deviation (*SD*), median (interquartile range, IQR), or *n* (%). Standardized mean differences were calculated between adherent and non-adherent participants.

### 3.1 Developing and optimizing adherence prediction models

The cohort was randomly partitioned 60:20:20 into training (*n* = 4,039), calibration (*n* = 1,008), and validation data sets (*n* = 1,008). Only the training and calibration data sets were used in developing the models, including cross-validation to select hyperparameters. Multiple multivariate imputation via chained equations using a random forest algorithm in the mice package was used to estimate missing data (Buuren and Groothuis-Oudshoorn, 2011; Li et al., 2015). Adherence as a continuous variable of the percentage use ≥4 h a night (%use) was the outcome. All other variables (features) were included in the model based on clinical and physiologic plausibility rather than variable selection via machine learning approaches for feature selection. Models tested included linear regression, least absolute shrinkage and selection operator (LASSO), Bayesian LASSO, elastic net, random forest, conditional inference random forest, gradient boosted machine (GBM), support vector machine (SVM) regression (both linear kernel and radial basis function kernel), spike and slab regression, and several neural network models—flat and multilayer neural networks (variable number and size of hidden layers, initialization weights, initial learning rates, and optimizer algorithms), model-averaged neural networks, Bayesian regularized neural networks, and neural networks with feature extractions. The caret package was used to cross-validate parameter estimates and optimize hyperparameter values using 10-fold cross-validation performed 4 times (Kuhn, 2008, 2019). A grid search was used to identify the model with a minimum cross-validated root mean square error (RMSE) (Kuhn, 2008, 2019). The model parameters tested are found in Table 1. These models were then recalibrated via the Dalton method (Dalton, 2013), using the calibration data set. Briefly, adherence was calculated for the calibration data set using the model developed in the training data set. The difference between the actual and predicted adherence was then calculated. A natural spline regression of the difference between actual and calculated adherence (dependent variable) vs. the predicted adherence (independent variable) to find offset parameters to calibrate the model. Model performance was evaluated in the validation data set with RMSE as the primary measure and mean absolute error (MAE) as the secondary measure. A calibration slope and intercept were evaluated from models fit on the validation data set to evaluate calibration (Alba et al., 2017).

**Table 1:** Model parameter tuned using a grid search.

**Model**	**Parameters tuned**
Linear regression	None
Ridge	Alpha
	Lambda
Elastic net	Alpha
	Lambda
Random forest	Mtry
	Minimal node size
Gradient boosted machine	Interaction depth
	Number trees
Shrinkage
Support vector machine with linear kernel	Cost
	Loss
Support vector machine with radial kernel	Sigma
	C
Naïve Bayes with LASSO	Sparsity
Naïve Bayes—spike and slab	Vars
Bayes regularized neural net	Neurons
Neuralnet	Layers sizes for 2 and 3 layers
	Threshold
AvNNet	Neurons
	Decay
nnet	Neurons
	Decay
pcaNNet	Neurons
	Decay
	Rang

### 3.2 Secondary analyses

We evaluated which group of variables had the highest impact on prediction performance. The best model was built on the training data set by removing from the main analysis groups of variables as follows: psychiatric medications, psychiatric comorbidity, cardiovascular comorbidity, pain comorbidity, sleep comorbidity, and tobacco and alcohol use/abuse. Analyses were evaluated in the validation data set with RMSE and MAE. In addition, we examined the effect of adding 30-day adherence data on model performance as this is a common time point to check in on therapy adherence. We also examined the models' performance on non-grouped (raw) data.

All analyses were conducted using R software version 3.4.3 (R Core Development Team, Vienna, Austria) (Core Team, 2014). This analysis used the mice, caret, glmnet, randomForest, and tidyverse packages extensively (Liaw and Wiener, 2002; Buuren and Groothuis-Oudshoorn, 2011; Simon et al., 2011; Fritsch et al., 2019; Kuhn, 2019; Wickham et al., 2019).

## 4 Results

### 4.1 Study population

Table 2 reports the baseline characteristics of the cohort. The 5,047 participants in the overall cohort were 38.3 ± 11.9 years old, and 3.9% female. The 90-day adherence distribution was bimodal with modes at 0% and 100% use >4 h/night; participant adherence at 90 days was 44.0% ± 40.4% (mean ± *SD*), with 36.9% of participants adherent by the Centers of Medicare & Medicaid Services standard of at least 70% of nights used at least 4 h a night. Among those who were adherent based on this standard, mean use >4 h/night was 91.2% ± 10.6%, whereas non-adherent individuals used PAP at least 4 h/night on 16.5% ± 21.6% of nights. The cohort had a high number of people with cardiometabolic comorbidity: 62.5% with obesity, 49.3% with diabetes, and 36.8% with coronary artery disease. In addition, there was notable psychiatric comorbidity as evidenced by 52.6% with depression, 29.6% with anxiety, and 25.0% with PTSD history. There was also substantial overlap in the psychiatric diagnoses, with 37.2% of the cohort with two or more diagnoses. The non-adherent participants were significantly younger (aged 37.9 ± 12.2 years vs. 40.4 ± 11.2 years) with more history of psychiatric comorbidity, alcohol abuse (24.5% vs. 13.8%), and tobacco use (40.1% vs. 29.6%). Non-adherent participants had a substantially increased use of benzodiazepines, antipsychotics, and mood stabilizers. Cardiac comorbidities were evenly balanced across groups.

**Table 2:** Baseline characteristics^*^.

**Characteristic**	**Overall (*n* = 5,047)**	**Adherent^†^ (*n* = 1,861)**	**Non-adherent^||^(*n* = 3,186)**	**SMD**
Age (years)	38.8 ± 11.9	40.4 ± 11.2	37.9 ± 12.2	**0.22**
Female gender	198 (3.9%)	59 (3.2%)	1,39 (4.4%)	0.06
**Comorbidities**				
Obesity	3,156 (62.5%)	1,207 (64.9%)	1,949 (61.2%)	0.08
Diabetes	2,489 (49.3%)	946 (50.8%)	1,543 (48.4%)	0.048
Heart failure	834 (16.5%)	282 (15.2%)	552 (17.3%)	0.06
Coronary artery disease	1,856 (36.8%)	682 (36.6%)	1,174 (36.8%)	0.004
Depression	2,656 (52.6%)	897 (48.2%)	1,759 (55.2%)	**0.14**
PTSD	1,261 (25.0%)	378 (20.3%)	883 (27.7%)	**0.17**
Anxiety	1,495 (29.6%)	491 (26.4%)	1,004 (31.5%)	**0.11**
Insomnia	776 (15.4%)	217 (11.7%)	559 (17.5%)	**0.17**
Alcohol abuse	1,037 (20.5%)	257 (13.8%)	780 (24.5%)	**0.27**
Tobacco use	1,826 (36.2%)	550 (29.6%)	1,276 (40.1%)	**0.22**
**Medication use**				
Benzodiazepines	1,088 (21.6%)	344 (18.5%)	744 (23.4%)	**0.12**
BZRA	376 (7.4%)	112 (6.0%)	264 (8.3%)	0.09
SSRI/SNRI	2,623 (52.0%)	899 (48.3%)	1,724 (54.1%)	**0.12**
Antipsychotics	1,024 (20.3%)	277 (14.9%)	747 (23.4%)	**0.22**
Mood stabilizers	803 (15.9%)	232 (12.5%)	571 (17.9%)	**0.15**

BMI, body mass index; BZRA, benzodiazepine receptor agonists; PTSD, posttraumatic stress disorder; SMD, standardized mean difference; SSRI/SNRI, selective serotonin reuptake inhibitor/serotonin norepinephrine reuptake inhibitor. ^*^Statistics presented as mean ± SD, median (25th percentile, 75th percentile), or *n* (%). ^†^Adherent was defined as positive airway pressure therapy use of at least 4 h a night for at least 70% of nights in the first 90 days after machine disbursement. ^||^Non-adherent was defined as using < 70% of night for at least 4 h a night in the first 90 days after machine disbursement. Bold indicates significantly different.

### 4.2 Adherence model performance

**Figure 2:**
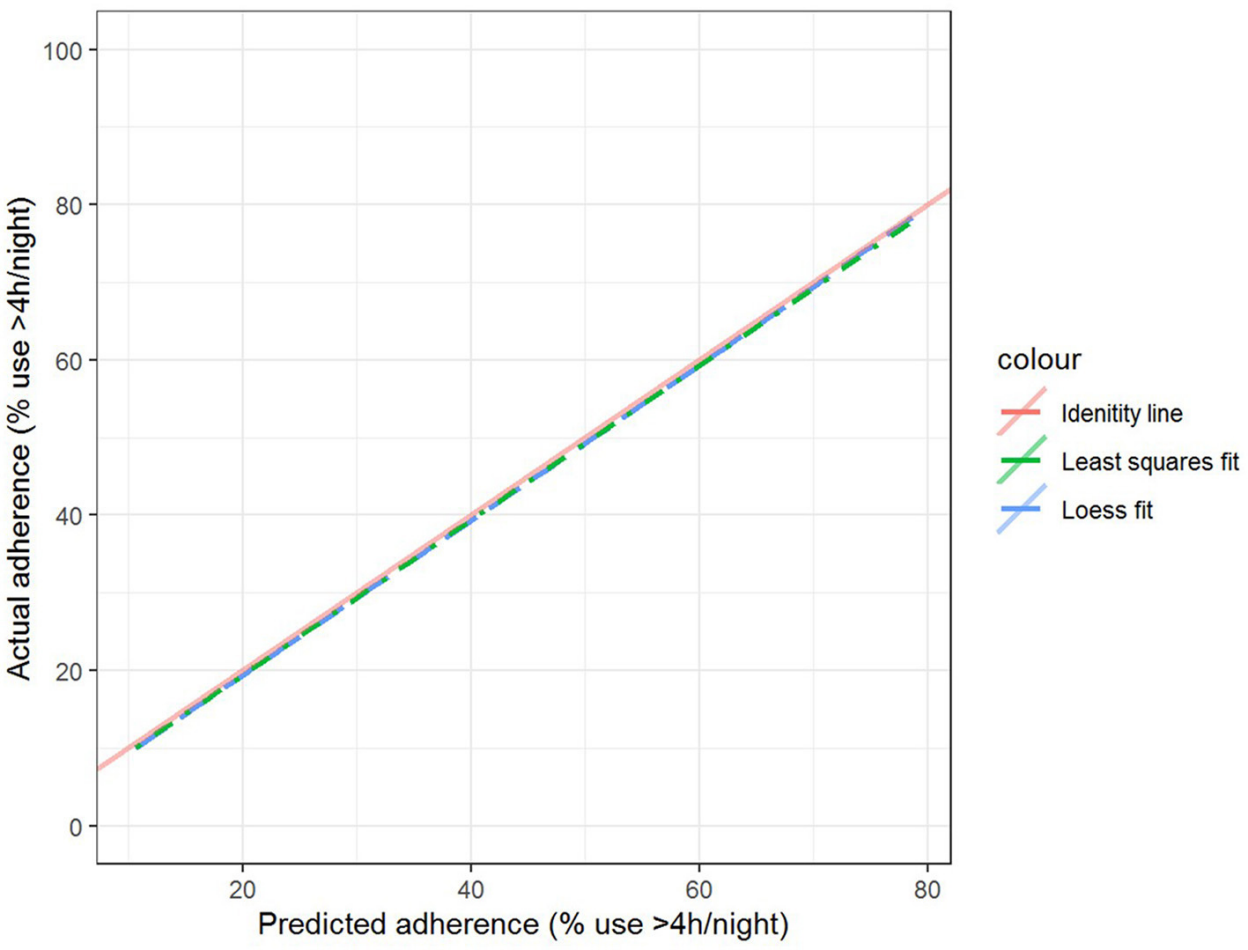
Calibration curves for gradient boosted machine model.

The best model after hyperparameter tuning was GBM, with an RMSE of 37.2 and an MAE of 33.1 (Table 3, Figure 2); this indicates that predictions by this model were off by 37% on average from the actual value. However, other models including the least sophisticated—cross-validated linear regression—were only slightly worse on this data set. The GBM performed the best in models that included 30-day adherence and PAP effectiveness information (Table 4). Models with 30-day PAP data performed substantially better than all other models. However, removing features (e.g., psychiatric comorbidity) did not substantially affect model performance (Table 5). Models that did not group variables (e.g., cardiac comorbidity vs. individual diagnoses of heart failure, coronary artery disease, etc.) but instead used each diagnosis performed better than those with grouped variables (RMSE 37.2 in GBM with grouped variables vs. RMSE 36 for the raw data model, including 102 features) (Table 6). Models that were restricted by eliminating variables with near-zero variance were not substantially worse than the full feature set (42 variables, GBM RMSE 36.1).

**Table 3:** Performance characteristics of machine learning algorithms for 90-day adherence in the validation cohort^*^.

**Adherence model**	**Root mean square error**	**Mean absolute error**
Linear regression	37.31	33.30
LASSO	37.32	33.27
Elastic net	37.31	33.27
Bayesian LASSO	37.36	33.28
Random forest	37.58	33.86
Conditional inference random forest	37.40	33.46
GBM	**37.21**	**33.07**
Spike and slab regression	37.32	33.31
SVM linear kernel	37.31	33.30
SVM radial kernel	37.38	33.35
Bayesian regularized neural network	37.34	33.33
Model averaged neural network	37.34	33.36
Neural network with feature extraction	37.52	33.51
Flat neural network	37.77	34.06

GBM, gradient boosted machine; LASSO, least absolute shrinkage and selection operator; SVM, support vector machine. ^*^Bold data indicate algorithm with lowest error.

**Table 4:** Performance characteristics of machine learning algorithms for 90-day adherence with additional 30-day adherence information in the validation cohort^*^.

**Adherence model**	**Root mean square error**	**Mean absolute error**
Linear regression	24.52	17.56
LASSO	24.49	17.52
Elastic net	24.50	17.53
Bayesian LASSO	24.54	17.57
Random forest	24.72	17.86
Conditional inference random forest	24.49	17.61
GBM	**24.43**	**17.46**
Spike and slab regression	24.52	17.55
SVM linear kernel	24.51	17.55
SVM radial kernel	24.54	17.48
Bayesian regularized neural network	24.55	17.58
Model averaged neural network	24.86	17.84
Neural network with feature extraction	24.68	17.76
Flat neural network	24.55	17.67

GBM, gradient boosted machine; LASSO, Least absolute shrinkage and selection operator; SVM, support vector machine. ^*^Bold data indicate algorithm with lowest error.

**Table 5:** Performance characteristics of GBM algorithm with and without various feature sets^*^.

**Adherence model**	**Root mean square error**	**Mean absolute error**
Original model	37.17	32.97
Original model with 30-day PAP data	**24.44**	**17.45**
No cardiac comorbidity	37.14	32.95
No psychiatric comorbidity	37.16	32.97
No psychiatric medications	37.16	32.98
No sleep comorbidity	37.22	33.01
No pain comorbidity	37.16	32.96
No alcohol/tobacco use/abuse	37.25	33.18

GBM, gradient boosted machine; PAP, positive airway pressure therapy. ^*^Bold data indicate algorithm with lowest error.

**Table 6:** Performance characteristics of GBM algorithm in models with individual diagnoses and medications (raw model), individual diagnoses and indications with near-zero variance features removed, and grouped diagnoses and medication classes^*^.

**Adherence model**	**RMSE**	**MAE**
Original model (grouped classes)	37.21	33.07
Original model with 30-day PAP data	24.43	17.46
Raw data model (102 variables)	37.21	33.07
Raw data model with 30-day PAP data (111 variables)	**24.27**	**17.25**
Raw model without NZV variables	37.07	33.00
Raw model with 30-day PAP data without NZV variables	24.30	17.27

GBM, gradient boosted machine; MAE, mean absolute error; NZV, near-zero variance; PAP, positive airway pressure therapy; RMSE, root mean square error. ^*^Bold data indicate algorithm with lowest error.

## 5 Discussion

This investigation compared several machine learning algorithms for predicting adherence to PAP therapy in a veteran population using robust methodologies. We found the GBM algorithm to be incrementally the most accurate model after hyperparameter tuning. However, other evaluated models performed similarly, and none was deemed clinically distinct at this point. Even though model performance overall was subpar, these models could be used to identify those people who are highly likely to be extremely adherent (and therefore need limited intervention) or not use their PAP at all (and, therefore, be candidates for alternative therapy). Adding 30-day adherence data substantially improved the accuracy of the predictions for all models; however, there is a high probability of data leakage because 30-day adherence is also included in the outcome, 90-day adherence. Additional model improvements would be expected if sleep study, symptom, and contextual factors were added to models. This is the first study to our knowledge to (1) try to predict adherence as opposed to finding associations with variables, (2) compare multiple machine learning algorithms using reproducible methods to evaluate a sleep condition, and (3) use data that were readily available from the EMR to make such predictions.

The next logical step to consider is whether incorporating other data improves predictive performance. Work in medication adherence has consistently shown that prior adherence to medications is a good predictor of future medication adherence (Muntner et al., 2014; Kumamaru et al., 2018; Zullig et al., 2019). Therefore, information on adherence to medication and no-show rates to scheduled medical visits may improve prediction accuracy. However, other variables, such as age, although strongly associated with adherence, had no ability to discriminate between who would or would not be adherent. Attitudes about therapy are associated with adherence and may aid in determining who will or will not be adherent (Balachandran et al., 2013). Perhaps the addition of a point-of-care questionnaire on attitudes before the start of PAP therapy could improve adherence prediction accuracy. Furthermore, integrating social determinants of health may improve prediction accuracy as shown in models of cardiovascular risk (Dalton et al., 2017).

Any adherence prediction model that is developed for VA data will need to be externally validated because there are inherent and dramatic differences between the VA population and the non-VA general sleep population. In the VA, there is a very high percentage of men, a non-representative racial mix, and much more psychiatric comorbidity. Hence, these findings may not be representative of EMR prediction algorithm performance in other populations. In particular, model discrimination can be lower when the risk profile of the analyzed population is relatively homogeneous, as may be the case with the population in this analysis.

Given the recent popularity of neural networks, it is perhaps counterintuitive that they were not able to accurately predict veterans' future adherence and that GBM—an ensemble of decision trees—had superior performance. However, neural networks are not a panacea. Not only is algorithm performance very dependent on the data and field for which it is being developed, but there is also evidence that tree-based models may be an improvement on neural networks in several situations (Fernández-Delgado et al., 2014). SVM outperformed neural networks in both binary classification for image identification and corporate bankruptcy prediction (Moghaddam and Yang, 2001; Shin et al., 2005) and multiclass prediction problems such as financial time-series forecasting and protein folding (Ding and Dubchak, 2001; Tay and Cao, 2001). Neural networks work best with large training sets in cases in which there are complex hierarchical relationships. In addition, there are numerous hyperparameters for neural nets, and hyperparameter value selection is complicated. Finding the ideal architecture by tuning the numerous hyperparameters inherent to neural networks is not intuitive, and even a grid search is not guaranteed to guide one in the optimal direction for hyperparameter optimization. Furthermore, neural networks are prone to finding local, rather than global, minima, and overfitting. This may be why when testing 179 algorithms in 121 data sets, tree-based methods were found to be the best family of models (Fernández-Delgado et al., 2014). In addition, the interpretability of models is crucial in medicine to detect bias and build trust in the model. Neural networks suffer from poor interpretability. Even small changes in parameters can lead to substantial shifts in model output. Because neural networks are often non-intuitive and opaque—with no clear explanation for why one set of variables slightly differing from another yielded substantially different results—using these models would be hard to justify in medicine in their current form. However, there is fervor in the community to develop tools to allow a better understanding of model decision-making (Teng et al., 2022). Despite the recent popularity of neural networks, they are not the best tool for every data analytic job.

Several study strengths and limitations are worth noting. This study used a separate training and validation data set (no part of the validation data set was used in any part of the training algorithm), which safeguards validity. The present study enrolled a broad cohort of every eligible patient who received a PAP machine, regardless of whether they used it or not. This large data set allowed advanced machine learning models to be used, such as neural nets, which smaller data sets would have difficulty converging. This expansive approach of gathering the entire clinical population given PAP therapy may improve performance in other clinical populations, at least within the VA. Furthermore, data included a limited subset of features that are readily gleaned from any EMR, giving the ability to implement such a method at scale in health systems. The evaluation of multiple machine learning algorithms allows for comparison in the validation data set. Several limitations are also worth noting. The cohort is from a VA population, which is largely male with a higher psychiatric and cardiac comorbidity; therefore, this veteran cohort may not be generalizable to the broader OSA population. This EMR data set misses potentially important predictors by not assessing patient symptoms or sleep study data. Medical treatment has evolved over the 10-year span that was sampled in this study and may affect adherence patterns. Additional bias in the data may incorrectly classify people because it lacks contextual data such as beliefs, socioeconomic status, and implicit bias in health care; this concern has higher prominence in data sets with limited diversity (e.g., low number of women in the present data set). If used for decision-making, further validation of such algorithms is paramount. It is notoriously difficult to understand the decision process of neural net models, and this is a detractor in medicine because the ability to explain the process is often important in understanding and improving the error rate.

Study findings could be broadened by examining populations enriched with women and minorities. Reevaluation and redevelopment of an adherence algorithm in larger, diverse cohorts may yet show the utility of these variables in determining future adherence. Future work may focus on identifying subgroups within the non-adherent population to guide the provision of enhanced interventions to promote adherence or encourage the use of a different treatment modality. Further refinement of adherence models with additional EMR data (e.g., anthropometry, physiologic variables, and past adherence to medications) may yield a more potent ability to predict future adherence. Adding contextual and social factors, such as marital status, presence of a bed partner, socioeconomic variables, and attitudes, could also improve prediction performance.

We were able to develop and calibrate a model for PAP adherence using EMR data. After evaluating several machine learning algorithms, including neural nets, we found GBM to be superior in predicting 90-day PAP therapy adherence; however, no model had sufficient performance for clinical application. Further work on refining the feature set to predict adherence is warranted. Improving the prediction of future adherence may open avenues for personalized therapy at OSA diagnosis.

## Data availability statement

The data analyzed in this study is subject to the following licenses/restrictions: adherence data is provided by Philips Respironics. VA Privacy Officer would need to review and clear all datasets before release. Requests to access these datasets should be directed to drannamay@gmail.com.

## Ethics statement

The requirement of ethical approval was waived by Louis Stokes Cleveland VA Medical Center Institutional Review Board for the studies involving humans because study is exempt per 45 CFR 46.104(d) (4). The studies were conducted in accordance with the local legislation and institutional requirements. The Ethics Committee/institutional review board also waived the requirement of written informed consent for participation from the participants or the participants' legal guardians/next of kin because retrospective chart review with minimal risk could not reasonably be done with informed consent.

## Author contributions

AM: Conceptualization, Formal analysis, Funding acquisition, Project administration, Resources, Writing—original draft, Writing—review & editing. JD: Conceptualization, Methodology, Supervision, Writing—review & editing.
